# Is hypernatremia worth its salt?

**DOI:** 10.1016/j.ccrj.2024.11.001

**Published:** 2024-12-06

**Authors:** Balasubramanian Venkatesh

**Affiliations:** The George Institute for Global Health, Gold Coast University Hospital, Australia

“Water, water everywhere nor any drop to drink”

The Rime of the Ancient Mariner by ST Coleridge

Sodium is the principal cation in the extracellular fluid, maintained at a concentration between 135 and 145 mmol/L, and is a major determinant of plasma tonicity. Plasma sodium concentration is a balance between the intake and losses of sodium and water.

In patients admitted to the Intensive Care Unit (ICU) with normal serum sodium (Na^+^) concentrations, hypernatremia (Na^+^ > 145 mmol/L) is reported to occur in about 10–15 % over the course of their ICU stay^1^^,^^2^. In critically ill patients, this may result from a) excess Na load as may happen secondary to administration of Na-rich fluids, hypertonic saline and sodium bicarbonate, or sodium-rich antibiotics such as ticarcillin sodium, b) administration of corticosteroids or c) renal or extra renal-free water losses – seen with diabetes insipidus, diuretic use, burns or diarrhoea. Hypernatremia is associated with neurological symptoms and with excess mortality.^1^^,^^2^

In this issue of CCR, two large retrospective cohort studies by Drs. Nasser et al. and Dr. Chaba^3^^,^^4^ et al. report the prevalence of ICU-acquired hypernatremia (ICU-AH), patient characteristics, trajectory, risk factors and outcomes. Both studies screened large numbers of ICU admissions (n = 55,255 and 11642). They excluded patients with a neurological, trauma, fulminant liver failure or post-cardiac arrest diagnosis, where hypernatremia might represent a therapeutic intervention aimed at decreasing cerebral oedema.

Drs. Nasser et al. reported a 7.5 % prevalence of ICU-AH (4.8 % mild hypernatremia, serum Na 145–150 mmol/L, 1.9 % moderate serum Na 150–155 mmol/L and 0.7 % severe-serum Na> 155 mmol/L). A high APACHE III score, invasive ventilation, fever, and diuretic use on the day before hypernatremia were independent risk factors for moderate or severe ICU-AH. After adjusting for confounders, all levels of hypernatremia were independently associated with mortality.

The prevalence of severe AH in the study by Chaba et al. was very similar – 0.9 %. This study also provided new insights – combined urinary electrolyte and free water clearance measurements in ICU patients and information on additional risk factors for AH and near-absent or limited or delayed water administration. About 28 % of patients with severe AH had delirium and the hospital mortality was 24 %.

These represent some of the largest cohort studies on hypernatremia and particularly highlight the low frequency of urinary electrolyte and free water clearance assessment in ICU patients, how little free water is administered to these patients and the slow rate of correction of serum sodium. Both studies make the argument for an interventional study for treating hypernatremia on the grounds that it is an independent predictor of mortality.

The results raise several important questions.

Is hypernatremia a direct contributor to adverse outcomes or is it merely an epiphenomenon occurring in the context of critically ill patients with multi-organ dysfunction? A review of the baseline characteristics and risk factors would suggest that in comparison to patients with normal serum Na, those with AH had higher catecholamine requirement and SOFA scores, were hyperlactatemic, a greater proportion were mechanically ventilated and predominantly medical admissions – and therefore likely to have higher mortality. These patients often tend to retain fluid which can impede ventilatory wean prompting clinicians to prescribe diuretic therapy with a view to achieving negative balance, the trade-off being hypernatremia.

What was not reported in either of the studies was corticosteroid usage – a recognized risk factor for AH^5^– often used in extremely sick patients with ARDS, septic shock, severe community-acquired pneumonia and obstructive lung disease – a group with high mortality. Despite the authors conducting detailed multivariate analyses to account for the various confounders, deciphering the precise contribution of hypernatremia to mortality in a retrospective dataset is challenging. Information on the causes of death would provide further insight into any potential causation. Of interest, in a study of patients with severe hyponatremia, following a comprehensive retrospective review of the medical records and causes of death, the authors concluded that patients died with and not due to the hyponatremia.^6^

The authors make a convincing argument for liberalising free water intake in critically ill patients with hypernatremia but why do ICU doctors appear to be reluctant to give enough water to severely hypernatremic patients? Many factors could contribute to this – first, ICU physicians are likely to recognise AH as an acceptable side effect of the therapies they have instituted such as diuretics and therefore correction of the Na^+^ concentration with free water might defeat the very purpose for which they initiated diuretic therapy. To correct a Na^+^ from 155 to 145 mmol/L in a 70 kg individual would require an additional 2.9 L of free water. Assuming this correction happens over a 48 h period, this would necessitate administering an extra 1.5L per day. There are some practical challenges in delivering this therapy. Administering this volume enterally would be challenging when gut function is often compromised in critically ill patients with the attendant risks of increasing nasogastric aspirates and pulmonary aspiration. Parenteral administration of free water is possible, and this could be achieved with either 5 % dextrose or hypotonic dextrose saline solutions or 0.45 % saline. However, these solutions come with an obligatory glucose/caloric load or a Na + load. Concern about prolonging ventilatory wean or worsening cardiac function with additional fluids may be another consideration.

The authors also make the case for a clinical trial of interventions for hypernatremia. Are we ready for a trial of tight sodium control? Many interventions are available including free water and thiazide diuretics to name a few.^7^^,^^8^ A clinical trial would be an important next step; however, it is not without its challenges. Firstly, characterising the true prevalence of hypernatremia is important. The analytical method, particularly indirect ion-sensitive electrodes (used in chemical pathology laboratories) can introduce measurement errors such as pseudohypernatremia (normal serum sodium is characterised as hypernatremia secondary to hypoproteinemia) or pseudonormonatremia (elevated serum Na characterized as normonatremia because of hyperproteinemia).^9^ Second, not all hypernatremic states are similar. The physiological milieu of hypernatremia from free water deficit would be different to that secondary to corticosteroid therapy for critical illness where there is likely to be excess body water. Third, sodium homeostasis in critically ill patients is complex and there may be no direct correlation between the volume of free water administration and the decline in serum Na^+7^. In this context, the role of Na + reservoirs such as bone and muscle in modulating serum Na in hypernatremia is not well understood.[10], [11] From a clinical perspective, at what serum Na level should correction begin – 150 or 155? Will clinicians have the equipoise to administer or withhold free water? Will liberalising free water administration come at the expense of longer duration of respiratory support? A well-conducted clinical trial would provide answers to these important questions.

## Conflict of interest

The authors declare that they have no known competing financial interests or personal relationships that could have appeared to influence the work reported in this paper.
